# Hospital Necker

**Published:** 1846-08

**Authors:** 


4. *Hospital Necker.—Clinical Lectures on Diarrhœa of Infants.*—By Professor TROUSSEAU.—The influence of the change of seasons is very strongly felt in hospitals destined to infants. During the course of the winter a numerous series of pulmonary and cephalic disorders has passed before us; we are entering now into spring, and the coming heat will bring with it intestinal affections. Soon, perhaps, you will not in one month meet, in these wards, with a single case of pneumonia, twelve or fifteen cases of which were admitted during every month of the winter. Thoracic diseases will be replaced by abdominal symptoms, and amongst these diarrhœa being the most frequent, the most difficult to treat properly, and the least understood, we will endeavour to prepare you beforehand for its observation by some remarks upon its pathology and treatment. The subject is extremely difficult, and although I have for eight years devoted myself to a daily study of the maladies of infancy, I feel myself in the dark with regard to many of its details, and do not therefore pretend to give you a completely satisfactory description, but merely to impart the little I do know of the matter. In order to introduce some regularity in the following remarks, we deem it necessary to establish a practical division between the various sorts of diarrhœa which are observed in children. We acknowledge only four primary forms of diarrhœa. 1, bilious diarrhœa; 2, mucous diarrhœa; 3, linteric diarrhœa; and 4, choleriform diarrhœa, or cholera infantilis. These forms are perfectly distinct from each other, and all the varieties of diarrhœa which may be observed in children, and which do not seem at first to have a place in our classification, will be found to consist of combinations of several of these original forms, or of deviations from these elementary types.

*Causes.*—Bilious diarrhœa may consist in a simple increase of the biliary and pancreatic secretions, or in a perversion of their qualities. Both may result from local irritation, but the first is often produced by mere physiological excitement. We will find a double illustration of these pathogenic influences, in the abundant flow of saliva determined by stimulation of the mouth with mercury, and in the increase of the secretion of tears caused by sorrow. Thus, slight inflammation of the stomach or duodenum will occasion a discharge of bile into the intestine; thus fear, anger, nervous excitement, in a word, will produce an increase of the biliary, pancreatic, and sometimes the renal secretions. We may say we meet with daily examples of the great power of physiological stimuli on the conglomerated glands. The diarrhœa of the young soldier who goes into action for the first time, is another common instance of the same kind, a further illustration of which we find in the influence of dreams on the spermatic organs. Violent exercise, abundant perspiration in many persons bring on diarrhœa. In the water-cure, a method of treatment too advantageous in some diseases to be entirely left to quacks, we find that during the process of packing, if the patient is made to drink several tumblers of water, abundant perspiration is thrown out, but if diaphoresis does not appear, the mucous surface of the intestine substitutes its action to that of the skin, and relieves the system by diarrhœa. One of the most frequent causes of diarrhœa will be found to reside in the quality of the food. The presence of globules of colostrum in the nurse's milk, due, as Donné has proved, to latent irritation of the mamma, sudden weaning, the exhibition of improper food, are all circumstances by which diarrhœa may be occasioned in the infant. The habit of covering the child in bed with too warm clothing, is also a frequent cause of disease. This is unfortunately a habit very prevalent among the lower classes in this country. So many as four blankets are thrown over the child, who is besides enveloped in swaddling clothes; and to add to the child's comfort, the mother not unfrequently adds her pillow to his other clothing. Abundant perspiration is thus produced, and, without any regard for the consequences, the child is extracted from his bed to be suckled or cleaned, thus being exposed several times a day to sudden changes of temperature, the result of which is pulmonary disease in winter, and intestinal derangement in summer.

*Semeiology.*—Bilious diarrhœa generally follows slight feverishness, and is often preceded or accompanied by vomiting. The mouth is bitter, the tongue foul, and the appetite absent. The colour of the motion varies from yellow to green, according as the biliary secretion is changed in its quality, or only increased in quantity. Its duration is from three to six days, and its termination favorable, unless the case is mismanaged. It is in fact a slight catarrhal condition of the mucous surface. Mucous diarrhœa is marked by the discharge of a new secretion from the bowels; it is often the consequence of the first variety of disease, or of indigestion. The nutriment acts as a foreign body upon the intestine, producing local irritation, and the excretion of a slimy mucus. This is a very common, and fortunately not very dangerous form. But when the irritation of the digestive tube is carried beyond certain limits, matters take a more serious aspect; enteritis sets in, and the products of inflammation are passed with the motions. Colitis occasionally makes its appearance, attended with intense pain, betrayed by cries uttered two or three minutes before the motions, with which a small quantity of blood is sometimes mixed, the dejections assuming a dysenteric character. When the small intestine alone is inflamed, our third form of diarrhœa, lientery, appears. In this variety the food passes unaltered through the digestive organs, and is recognisable in the dejections, in which grains of rice, vegetable substances, curdled milk, can be readily distinguished. This is an extremely dangerous derangement, on account of the impossibility of refection.

After one of the above kinds of diarrhœa, occasionally without them, the cholera of children—that almost invariably fatal affection—is observed to show itself. After dejections of a bilious or mucous character, the infant is suddenly seized with violent vomiting, against which the efforts of art remain unavailable. A watery diarrhœa of a greenish hue is at the same time discharged from the bowels, and alarming general symptoms are noticed. The eyes sink in the orbits, the features are decomposed, the complexion becomes livid, and the nose, tongue, extremities, and even the breath, grow cold; the cry is acute, small, and incessant; the skin loses its elasticity, and when pinched in any part of the body, retains the folds made by the fingers, as if it were but an inert membrane. The child is sleepless, but without convulsions. Such are the first symptoms of this formidable malady. In its second period the vomiting, and sometimes the diarrhœa, cease, but no amendment follows. The collapse increases, and the infant almost invariably dies. We have, however, occasionally had the consolation of saving some few cases; one is at present in the wards, to whose case we called your attention, and who owes his recovery, under Providence, to the double tartrate of soda and potass.

*Treatment.*—We have found few drugs of any avail in the treatment of the bilious diarrhœa in children. It is a convenient plan to call the disease a gastro-enteritis, because the denomination leads to an invariable line of treatment, accessible to understandings of the meanest capacity. We take, however, a different view of diagnosis generally, and deem it unprofitable unless it leads to some practically useful indication. Some forms of diarrhœa are doubtless less difficult of cure than others, but we must say that the varieties we have described often combine with each other, so as to cause the practitioner no small embarrassment, and to reduce him, in many cases, to a blindfold empiricism; not but that we profess much respect for that empiricism which teaches us to exhibit mercury in syphilis, steel in anemia, and bark in ague; but the empiricism we deprecate as a contemptible method, is that which is not guided by diagnosis. The method we refer to may become a useful guide to the detection of the nature of disease, and it then acquires a considerable degree of utility. Let us remind you of a case of hemicrania at present in the wards. The attacks were periodical, and we tried sulphate of quinine without success; thus acquiring the knowledge that it was not governed by miasmatic influence. We exhibited then mercurial preparations, and the nervous headache having yielded at once, we were led to attribute the disease to syphilis. This is the empirical method we adopt; it is not the empiricism of *experiment*, but of *experience*. Thus, if we say that a patient is affected with neuralgia, we express a diagnostic opinion which is as elementary, and, let us add, as useless, as to say that he is affected with a corn on his foot; but it is quite another sort of thing to say that the patient is labouring under gouty, syphilitic, miasmatic, rheumatic, or chlorotic neuralgia, because this kind of diagnosis leads us to the real therapeutic indications. To return to the treatment of diarrhœa: Let us not forget that, to arrest the superabundant intestinal secretion is not by any means to cure the complaint which caused it. It is our opinion that bilious diarrhœa is only a very superficial catarrhal derangement of the intestine. The most efficient treatment consists in the exhibition of neutral salts, such as the double tartrate of potass and soda, phosphate of soda, Epsom or Glauber salts. We do not wish you to understand that we recommend the use of purgatives. No; castor oil and magnesia, or manna, you will usually find unsuccessful, whereas the neutral salts generally produce a speedy amendment. We have also derived benefit from the exhibition of the pulv. ipecac., at doses varying from two to ten grains, and mixed with a little jam, milk, or simple syrup. The action of this medicine is threefold; it is a substitutive, a discutient, and being a diaphoretic deviates towards the skin those vital energies which are occupied in the production of morbid symptoms in the alimentary canal. But when the bilious diarrhœa is the consequence of mere nervous excitement—when it is caused by fear or anger, as tears by grief, or salivation by appetite—opium gives relief in a very short time. In these cases, which you will find to be characterised by the absence of any sort of suffering during the first twenty-four or thirty-six hours, the disease will speedily yield to the influence of hypnotic medicines. Half a drop of Sydenham's laudanum is a sufficient dose for a child under six months; others, it is true, will bear two or three drops, but that dose is too powerful a narcotic for the many. The laudanum should be dissolved in an ounce mixture, whereof the patient shall take a teaspoonful every three or four hours; but when the disorder has lasted beyond the specified time, opium ceases to possess its salutary effect, because the mere presence of the increased secretions on the mucous surface has sufficed to bring on an irritation which did not exist at first. Then we must again have recourse to neutral salts.

In mucous diarrhœa we have generally derived benefit from three sources: saline purgatives, calomel, and rhubarb. The dose of calomel we recommended is one-fifth of a grain daily, mixed with half a drachm of sugar. This should be continued two or three days at furthest. As to rhubarb, it is the "syrup" we use, the so-called "sirop de chicorée"—a good preparation in everything but its absurd name, which insinuates the idea of the efficacy of the endive, which is on the contrary perfectly, inert. Great attention should be paid to the child's diet; his food, less abundant than usual, should be chosen with great care. Milk is the most proper food for young children; fecula and broth also may be given after the expiration of the first year, and the drink should be in small quantity. In the choice of food the physician must also allow himself to be guided, in a great measure, by the idiosyncracy of the child, and the mother's remarks on the peculiarities of his appetite. —*London Medical Times.*

